# LPV Control and Virtual-Sensor-Based Fault Tolerant Strategies for a Three-Axis Gimbal System

**DOI:** 10.3390/s22176664

**Published:** 2022-09-03

**Authors:** Ariel Medero, Vicenç Puig

**Affiliations:** Institut de Robòtica i Informàtica Industrial (CSIC-UPC), Llorens i Artigas, 4-6, 08028 Barcelona, Spain

**Keywords:** LPV, PID, LMIs, virtual sensor, gimbal, robotics, mechanical modelling, fault-tolerant control

## Abstract

This paper deals with the LPV control of a three-axis gimbal including fault-tolerant capabilities. First, the derivation of an analytical model for the considered system based on the robotics Serial-Link (SL) theory is derived. Then, a series of simplifications that allow obtaining a quasi-LPV model for the considered gimbal is proposed. Gain scheduling LPV controllers with PID structure are designed using pole placement by means of linear matrix inequalities (LMIs). Moreover, exploiting the sensor redundancy available in the gimbal, a virtual-sensor-based fault tolerant control (FTC) strategy is proposed. This virtual sensor uses a Recursive Least Square (RLS) estimation algorithm and an LPV observer for fault detection and estimation. Finally, the proposed LPV control scheme including the virtual sensor strategy is tested in simulation in several scenarios.

## 1. Introduction

Gimbals are precision devices used for orientation control of a second device in a 3D space. They can be utilized with mounted cameras to maintain horizontal orientation while being hand-held or even mounted on a helicopter or planes. They are also widely employed for hardware-in-the-loop tests for sensor technology, from testing smartphone gyroscopes and accelerometers to even test mission-critical navigation devices for future spacecraft. Despite their importance, industry still relies on the use of the classical PID controllers for the positioning control of the gimbal axes.

### 1.1. Related Literature

In the literature, PID-based controller solutions have also been proposed for gimbal systems. In [1], the authors propose a disturbance observer in conjunction with a classical PID cascade scheme for angular position tracking of the gimbal axes to enhance the low accuracy and bad disturbance rejection that affect PID controllers. A different approach to PID control of gimbal systems is applied in [2]. In this work, the authors compare the performance of PIDs tuned by using evolutionary algorithms, such as Particle Swarm Optimization and Genetic Algorithms, with traditional tuning methods, such as Ziegler–Nichols, showing an improvement of the evolutionary methods over the more traditional ways of tuning the PID controller parameters. In addition to PID controllers, some works for the control of gimbal systems have also focused on the use of nonlinear controller design. In [3], the use of a sliding mode controller is proposed for a two-axis gimbal system to deal with the high nonlinearites of such a system and the lack of model accuracy. This gimbal system is applied to a planar antenna in which both yaw and pitch angles are required to be controlled. On the other hand, Ref. [4] proposes using an adaptive control law that guarantees stability using the Lyapunov theory, with positive results both in simulation of a full three-axis system and experimental tests of a single-axis in the case of three-axis motion simulator used for test and calibration of spacecraft instrumentation. The same authors enhance their previous work in [5] by adding a reference model in the adaptive control law to improve the transient tracking performance. Thus, their controller falls within the category of model-reference adaptive control (MRAC), and additionally, they implemented nonlinear observers to estimate the accelerations of the gimbal axes and derivatives of the actuator motor currents which are required in their developed adaptive control law. Another approach for the control of such systems is the use of optimal control. In [6], the authors implement a linear quadratic regulator (LQR) control law for a three-axis motion simulator. They achieved this by linearizing the nonlinear model of the system using Taylor linear expansions at each time step and computing the varying optimal state feedback controller gain $K_{o}\left(t\right)$ that minimizes a given cost function. In [7], the authors use an optimization based MPC which uses a nonlinear third order Hammerstein model obtained from input–output data of a three-axis camera gimbal system mounted on a UAV drone. A comparison is also given of the proposed MPC with a PID controller tuned using the Ziegler–Nichols method proving the superiority of the former. Another line of control theory that has been proven to be successful when applied to real experimental situations is the Linear Varying Parameter (LPV) control theory [8]. It has been applied to the control of robotic manipulators, which are robotic systems quite similar to gimbal systems when only three axes are considered. In the case of [9], the authors embed the nonlinearities of the robotic manipulator in a quasi-LPV model and are able to use the most efficient Quadratic Programming approach to solve a quasi-LPV MPC problem instead of the most computationally intensive Nonlinear MPC one, allowing highly nonlinear systems to benefit from fast MPC approaches. Additionally, in [10], the authors use the polytopic LPV approach to compute a gain-scheduled state feedback controller using Linear Matrix Inequalities (LMI) methods to synthesise the controller using $H_{\infty }$ theory as well as *D*-stability for pseudo pole-placement for nonlinear systems. This allows extrapolating very powerful and well known controller synthesis strategies from Linear Time Invariant (LTI) systems to the nonlinear control. However, to the best of the authors’ knowledge, no LPV formalism has been applied to the control of three-axis gimbal systems.

### 1.2. Paper Contribution and Structure

The benefits of the LPV control theory for nonlinear systems and the ease of implementation of the final control law for the case of the polytopic LPV approach are the main motivations of this paper in order to apply them to the case of a three-axis gimbal system. In this paper, a systematic approach to develop control systems for gimbal setups based on nonlinear techniques is presented. The mathematical model of the three-axis gimbal is obtained following the well-known Serial-Link (SL) object theory from the robotic arm manipulators field using the Lagrange formulation. The controllers are based on LPV approaches for the design of robust PID controllers against the nonlinearities of the mechanical gimbal system model equations and disturbances that are expected to appear as non-considered Coulomb frictions or changes to the gimbal system due to the mounted external device. In fact, three alternative ways in which to design PID LPV controllers are presented, all of which take full advantage of the information of the nonlinear model in contrast to classical PID controller design where linearization is required. The efficiency and correctness of the developed control systems are tested using simulation results.

**Figure 1:**
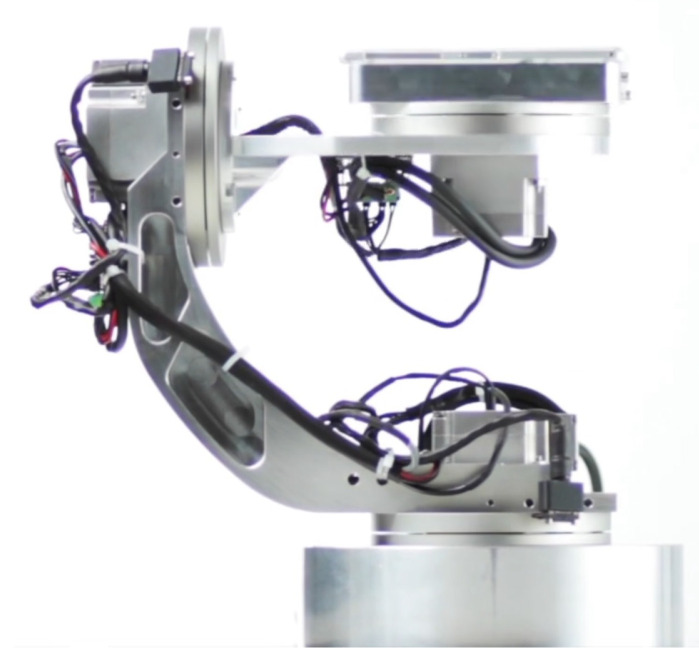
Photo of the three-axis gimbal.

As a reference for this paper, the three-axis gimbal shown in Figure 1, which is used as a camera mount to achieve accurate orientation of the images being recorded, is utilized. To accomplish this, this device presents three moving components that provide it with a full degree of freedom for angular positioning in the 3D space. Each independent moving part is driven by an AC servo actuator electric motor which includes an internal incremental encoder. In addition, in each joint of the gimbal, it counts with an absolute encoder. The incremental encoder allows a direct calculation of the angular speed of the gimbal links while the absolute encoder allows for the direct measurement of the angular position. Both encoders interface with a servodrive which takes care of the electric motor power management. So, the controller system design in this paper only considers the mechanical aspect of the three-axis gimbal control.

The search for the robustness of the controlled system serves as the motivation to exploit the sensor redundancy present in the three-axis gimbal and present a fault tolerant control (FTC) strategy to prevent malfunctions in the control system in case of a partial sensor failure. This method is based on the real-time estimation of possible faults parameters affecting the sensors by using LPV state observers and Recursive Least Squares (RLS) estimation methods in order to build a virtual sensor.

The main contributions of this work are then:

- To develop a quasi-LPV modelling approach for three-axis gimbals based on a detailed physical model obtained as a sequence of rigid bodies (links) interconnected by articulations (joints).
- To propose an LMI-based control design methodology that allows for quasi-LPV Gain-scheduling PID controllers for three-axis gimbals.
- To apply a virtual sensor strategy in a three-axis gimbals systems that provides fault-tolerant capabilities in case of partial sensor faults.

The paper is organized as follows. Section 2 and Section 3 are, respectively, concerned with the derivation of an analytical model for the gimbal in a systematic approach and the simplification of such models to obtain polytopic qLPV models for subsequent controller synthesis. The procedure to compute the controller gains is shown in Section 4, with emphasis on the model augmentation that allows obtaining gain-scheduled PID controllers and the LMIs tools that allow achieving pole-placement type of specification for nonlinear systems in a very formal and methodical way. Finally, Section 5 presents a Virtual Sensor algorithm that exploits the sensor redundancy, which exists in the used reference gimbal and in many industrial robotic manipulators, in order to provide robustness against partial sensor faults.

## 2. Modelling of the Gimbal

To obtain the mathematical representation of the three-axis gimbal dynamics, the well developed theory from the robotic manipulators field was used. Modelling the gimbal as a serial-link manipulator [11] offers a straightforward methodology to achieve a highly detailed model of the system as a sequence of rigid bodies (links) interconnected by means of articulations (joints). The first step in modelling a system as a serial-link object is to obtain an adequate set of reference frames (joint coordinate frame) with respect to each of the moving bodies and then compute the parameters that allow relating all the consecutive pairs of links that form the manipulator. The problems just described are part of the Direct Kinematics (DK) problem, which in the serial-link objects theory is solved by following the steps from the Denavit–Hartenberg (DH) Convention.

**Figure 2:**
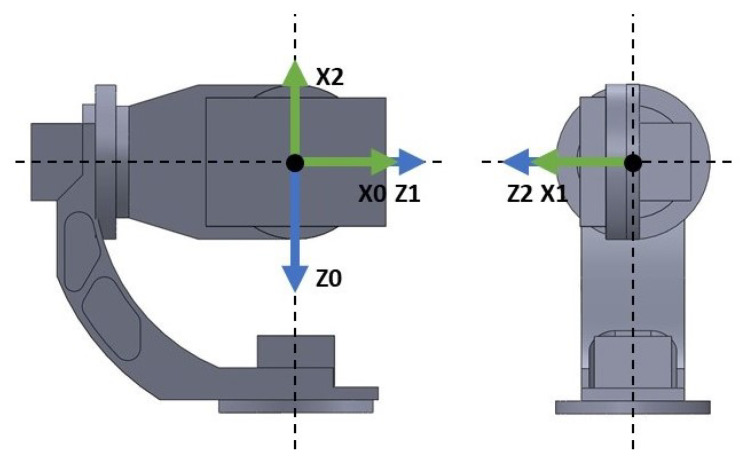
Axes of the joint coordinate frame.

Figure 2 shows the reference frames chosen for the reference gimbal as a result of following the DH Convention. Notice that all reference frames share a common origin; this has been performed to exploit a unique design feature of gimbals, which is that all three axis of rotations intersect at one point. By selecting the intersection point as the origin for all references frames, we simplify the DK by neglecting the translations of the links and solely focusing on the joints rotation. With that selection of reference frames and knowledge of the gimbal physical construction, a series of parameters, known as the DH parameters, can be computed. The list of specific DH parameters for the reference three-axis gimbal can be seen in Table A1. The DH parameters are used to obtain the Homogeneous Transformation matrix (1) which relates link i−1 with respect to link *i*. Finally, by the concatenation of the Homogeneous Transformation matrices, as in Equation (2), the DK of the gimbal $T_{0}^{3}\left(q\right)$ is derived, which for a given joint angles vector $q={\left[q_{1},q_{2},q_{3}\right]}^{T}$ allows us to know the 3D pose of the system in world coordinates. This 3D pose is obtained as a rotation matrix $R_{0}^{3}\left(q\right)$ which can be extracted from the Homogeneous Transformation of the DK. Equation (3) shows the structure of the DK for gimbals using a common origin for all reference frames as previously proposed.

(1) $$A_{i-1}^{i}=\left[\begin{array}{cccc} \cos\theta_{i} & -\sin\theta_{i}\cos\alpha_{i} & \sin\theta_{i}\sin\alpha_{i} & a_{i}\cos\theta_{i}\\ \sin\theta_{i} & \cos\theta_{i}\cos\alpha_{i} & -\cos\theta_{i}\sin\alpha_{i} & a_{i}\sin\theta_{i}\\ 0 & \sin\alpha_{i} & \cos\alpha_{i} & d_{i}\\ 0 & 0 & 0 & 1 \end{array}\right]$$

(2) $$T_{0}^{3}\left(q\right)=A_{0}^{1}\left(q_{1}\right)A_{1}^{2}\left(q_{2}\right)A_{2}^{3}\left(q_{3}\right)$$

(3) $$T_{0}^{3}\left(q\right)=\left[\begin{array}{cc} R_{0}^{3}\left(q\right) & 0\\ 0 & 1 \end{array}\right]$$

**Figure 3:**
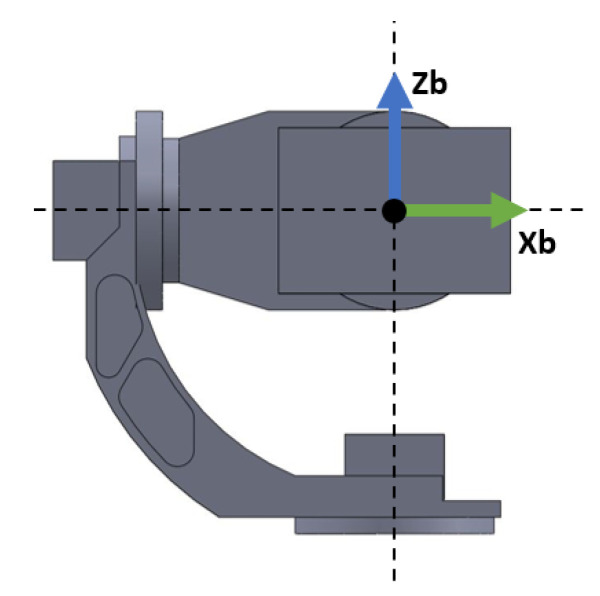
Base reference frame fixed to the three-axis gimbal base.

**Figure 4:**
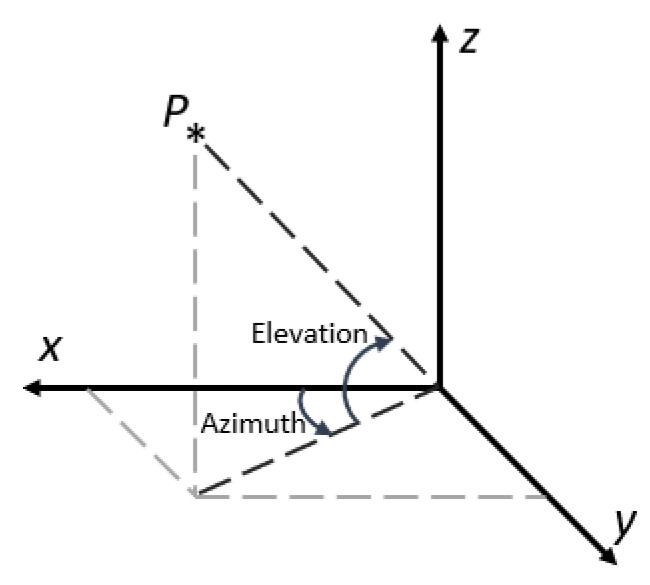
Azimuth and elevation angles definition.

The opposite problem to the DK is the Inverse Kinematics (IK) problem, which deals with obtaining the gimbal joint angles $q_{i}$ that allow achieving a specific 3D pose orientation. The IK problem is of interest in the case where a reference path is given in world coordinates, and as a result, it needs to be translated to joint coordinates for use as reference in the control system. First, note from Figure 2 that axis $z_{0}$, in solidarity with Link 1 is pointing downwards. This decision in motivated to have the reference axis coincide with the positive axis of rotation of the AC servo motor actuator for that link. As the motivation of the modelling phase is to obtain a model suitable to a control-oriented model, it is best practice to select axes positive with our actuators so that no adjustment needs to be made to the control signal computed from the controllers. For translating from the 3D world coordinates to the joint space coordinates, however, it is more natural to work with the base reference frame of Figure 3. This base frame is fixed to the three-axis gimbal base; as such, it does not have any degree of freedom. The DH parameters relating the base frame with the reference frame for Link 1 are given in Table A2. To compute the joint angles *q* that would allow them to point the gimbal to any world coordinate given in the base frame is a problem closely related to the the spherical transformation, as shown in Figure 4.

Then, the solution for the inverse kinematics problem for $q_{1}$ and $q_{2}$ is

(4) $$q_{1}=-Azimuth=-atan2\left(y,x\right)$$

(5) $$q_{2}=-Elevation=-atan2\left(z,\sqrt{x^{2}+y^{2}}\right)$$

The angle of rotation for Link 3 (for the reference gimbal) is only used to control the angle of rotation of the mounted camera with respect to the ground horizontal plane. So, its IK can be given simply by:

(6) $$q_{3}=-Tilt$$

With the Direct Kinematics of the gimbal derived, the following step is to derive the equations of motion of the three-axis gimbal. The dynamic model follows the classical Newton’s Law of motion, represented in Equation (7) in the joint coordinate frame rather than the Cartesian frame, as the former is more naturally related to the control problem.

(7) $$B\left(q\right)\ddot{q}+C\left(q,\dot{q}\right)\dot{q}+F_{v}\dot{q}+G\left(q\right)=\tau$$

where $q\in R^{3}$ represents the vector of joint angles $q={\left[q_{1},q_{2},q_{3}\right]}^{T}$, being $q_{i}$ the joint angle of the *i* link. $B\left(q\right)\in R^{3\times 3}$ is the global inertia tensor of the gimbal and is a symmetrical matrix, $C\left(q,\dot{q}\right)\in R^{3\times 3}$ is the Coriolis effect matrix, $F_{v}\in R^{3\times 3}$ is a diagonal matrix containing the constants for the viscous friction that affects each link, $G\left(q\right)\in R^{3}$ is the vector of external torques caused by the influence of gravity, and $\tau \in R^{3}$ represent the vector of external torques caused by the AC Servos acting on the joints and serve as the considered control input.

The mathematical representation for most of these terms can be obtained by following the Lagrangian formulation for serial-link objects, which is quite systematic and gives closed form equations for systems that could be modeled as robotic manipulators, as is the case of the gimbal. The equation to compute the inertia tensor matrix B(q) is

(8) $$B\left(q\right)=\sum_{i=1}^{3}\left(m_{l_{i}}J_{P}^{{\left(l_{i}\right)}^{t}}J_{P}^{\left(l_{i}\right)}+J_{O}^{{\left(l_{i}\right)}^{t}}R_{0}^{i}I_{l_{i}}^{i}R_{0}^{i^{t}}J_{O}^{\left(l_{i}\right)}\right)$$

The elements $J_{P}^{\left(l_{i}\right)}$ and $J_{O}^{\left(l_{i}\right)}$ are the translational Jacobian of the center of mass and rotational Jacobian for the *i* link given by

(9) $$J_{P}^{\left(l_{i}\right)}=\left[\begin{array}{cccccc} J_{P_{1}}^{\left(l_{i}\right)} & \cdots & J_{P_{i}}^{\left(l_{i}\right)} & 0 & \cdots & 0 \end{array}\right]$$

(10) $$J_{O}^{\left(l_{i}\right)}=\left[\begin{array}{cccccc} J_{O_{1}}^{\left(l_{i}\right)} & \cdots & J_{O_{i}}^{\left(l_{i}\right)} & 0 & \cdots & 0 \end{array}\right]$$

The columns for the translational Jacobian can be computed as:

(11) $$J_{P_{j}}^{\left(l_{i}\right)}=z_{j-1}\times p_{l_{i}}$$

where

(12) $$z_{j-1}=R_{0}^{j-1}z_{0}$$

(13) $$z_{0}={\left[\begin{array}{ccc} 0 & 0 & 1 \end{array}\right]}^{T}$$

(14) $$p_{l_{i}}=R_{0}^{i}R_{i}$$

Notice that $R_{0}^{i}$ represents the concatenation of rotational matrices from Link 1 to Link *i*, analog to (2), and $R_{i}$ are the Cartesian coordinates of the center of mass of Link *i* with respect to its own reference frame. The columns for the rotational Jacobian can be computed as:

(15) $$J_{O_{j}}^{\left(l_{i}\right)}=z_{j-1}$$

With the global inertia tensor matrix B(q) of the gimbal derived, then obtaining the $c_{ij}$ elements of the Coriolis matrix $C(q,\dot{q})$ is quite straightforward

(16) $$c_{ij}=\sum_{i=1}^{3}c_{ijk}{\dot{q}}_{k}$$

(17) $$c_{ijk}=\frac{1}{2}\left(\frac{\partial b_{ij}}{\partial q_{k}}+\frac{\partial b_{ik}}{\partial q_{j}}+\frac{\partial b_{jk}}{\partial q_{i}}\right)$$

Note that $b_{ij}$ are the elements from the global inertia tensor matrix B(q). The last element that the Lagrange formulation provides for serial-link objects is the vector of external torques in the manipulator joints due to the effect of gravity $G\left(q\right)=[g_{1}\left(q\right),g_{2}\left(q\right),g_{3}\left(q\right)]$, where the individual torques $g_{i}\left(q\right)$ can be computed as

(18) $$g_{i}\left(q\right)=\sum_{j=1}^{3}-m_{l_{j}}g_{0}J_{P_{i}}^{l_{j}}$$

(19) $$g_{0}={\left[\begin{array}{ccc} 0 & 0 & g \end{array}\right]}^{T}$$

Note that $g_{0}$ must be defined with respect to the Link 1 reference frame, which as can be seen in Figure 2 for the reference gimbal is pointing downwards, thus the gravity acceleration is positive in this particular coordinate frame.

In order to make the Simulation Oriented Model (SOM) more realistic, the equations of motion of the gimbal have been modified as in Equation (20) to include the effects of the static and kinetic Coulomb frictions in the model [12]

(20) $$B\left(q\right)\ddot{q}+C\left(q,\dot{q}\right)\dot{q}+F_{v}\dot{q}+G\left(q\right)+F_{c}=\tau$$

where

(21) $$F_{c}=\left\{\begin{array}{cc} F_{ext} & \mathrm{if}\;\dot{q}=0\;\mathrm{and}\;F_{ext}\leq F_{s}\\ \mu_{k}F_{s}\cdot \mathrm{sign}(\dot{q}) & \mathrm{if}\;\dot{q}\neq 0 \end{array}\right.$$

and the vector of external forces is the result of the effects of the actuators and the torque due to gravity

(22) $$F_{ext}=\tau -G\left(q\right)$$

## 3. Control-Oriented Model

The model equations obtained by following the Lagrangian method for serial-link objects are quite complete and close to the real system dynamics, especially with the addition of the Coulomb frictions. Thus, it is a very good SOM model. However, for controller synthesis, its equations are too complex and cumbersome to work with. In addition, the hard nonlinearities dependent on the link velocity introduced by the Coulomb friction make that model of the system behave as a hybrid nonlinear system rather than a simpler standard nonlinear system. For this reason, the Control Oriented Model (COM) to be developed is based on the representation given by (7), leaving the Coulomb frictions as unknown disturbances.

In spite of leaving the effect of the Coulomb frictions as disturbances, the model obtained from the Lagrangian method is still too complex for controller synthesis, so a series of simplifications are required. To gain insight into the system dynamics and attempt to understand which simplifications could be applied, a series of open-loop simulations have been carried to the full Lagrangian model without the Coulomb frictions effect.

**Figure 5:**
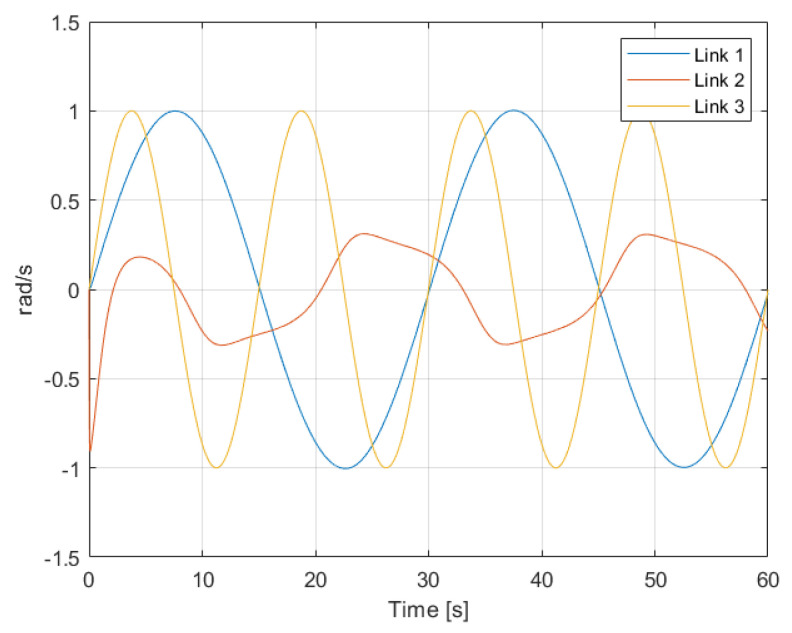
Angular velocity $\dot{q}$ of the links in the presence of a sinusoidal torque input.

**Figure 6:**
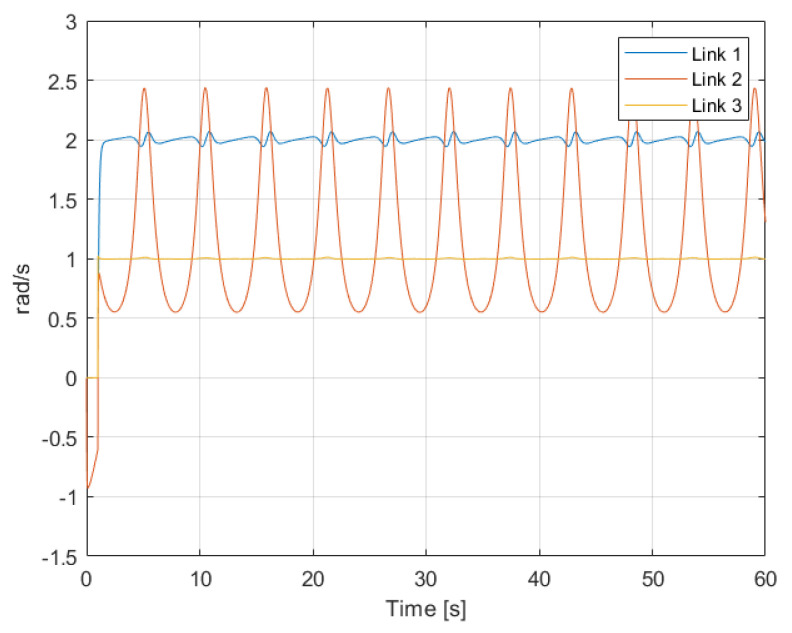
Angular velocity $\dot{q}$ of the links in the presence of a constant torque input.

From these simulation results, some important insights can be gathered. From classical control theory, it is known that the output response of linear systems to sinusoidal inputs is another sinusoidal input as well, but with different amplitude gain and angular phase. Analysing Figure 5, it can be seen that in the presence of an open-loop sinusoidal torque input, the angular velocity response of Links 1 and 3 is also pretty sinusoidal. That output response to sinusoidal inputs demonstrates that for those links, despite their dynamics being given by complex nonlinear equations, their dynamics are actually dominated by linear phenomena. The response for Link 2, on the other hand, clearly shows nonlinear behaviour in comparison. This nonlinearity can also be observed in the results from Figure 6. In the presence of a constant torque input, the angular velocity of Link 2 presents an oscillatory response caused by the effect of gravity in relation to the link’s angular position. In the other two links, which are not affected that much by gravity, the angular velocity is constant and with not much disturbance caused due to the erratic oscillations of Link 2.

From this quick analysis, two conclusions can be reached that justify model simplifications that can be applied to most three-axis gimbal systems. First, the dynamics of the links behave quite independently of each other, so there is a low level of coupling in the overall system. Second, the system showed to have an important linear behaviour, so the dynamics are not too dependent on the gimbal pose and changes in the links configuration for the most part. Then, two important simplifications can be made to the model: Firstly, we decouple the inertial tensor matrix B(q) and the Coriolis effect matrix $C(q,\dot{q})$ so that only the diagonal elements of each matrix are considered in the new $\tilde{B}\left(q\right)$ and $\tilde{C}\left(q,\dot{q}\right)$ matrices. Secondly, since the variation of the inertial configuration does not have a great impact on the system dynamics, we consider only the mean value for each of the elements of the diagonal of the $\tilde{B}\left(q\right)$ matrix so that the decoupled inertia tensor matrix is a constant diagonal matrix $\bar{B}$. After these simplifications, the COM equations can be easily rearranged as shown in (23), which is an appropriate representation of the LPV modelling approach

(23) $$\ddot{q}={\bar{B}}^{-1}\left[\tau -\tilde{C}\left(q,\dot{q}\right)\dot{q}-F_{v}\dot{q}-g\left(q\right)\right]$$

For the synthesis of the controllers, polytopic quasi-LPV models will be used for each gimbal link. The LPV methodology has several advantages with respect to other nonlinear control methods. Mainly, it allows representing the model as a pseudo-linear system, and as result it allows powerful and well studied techniques from the classical linear control design to be applied to our nonlinear COM. For the polytopic quasi-LPV modelling, two steps are required [13]: the nonlinear embedding followed by the polytopic representation [14].

The objective of the nonlinear embedding is to represent the COM of (23) as a pseudo-linear state space model, as in (24). Note that this system representation is independent for each *i* link. Exploiting the low coupling shown by the gimbal model, three independent models are derived, one per each link. Also note that the *A* matrix from the state space model is not constant as it depends on the angular link position and the angular velocity of the link. This pseudo-linear representation for each of the three links of the gimbal have as a generic representation the one shown in (26)

(24) $${\dot{x}}_{i}=A_{i}\left(q,\dot{q}\right)x_{i}+B_{i}\tau_{i}$$

where

(25) $$x_{i}=\left[\begin{array}{c} q_{i}\\ {\dot{q}}_{i} \end{array}\right]$$

(26) $$\left[\begin{array}{c} {\dot{q}}_{i}\\ {\ddot{q}}_{i} \end{array}\right]=\left[\begin{array}{cc} 0 & 1\\ \theta_{j}\left(q,\dot{q}\right) & \theta_{j+1}\left(q,\dot{q}\right) \end{array}\right]\left[\begin{array}{c} q_{i}\\ {\dot{q}}_{i} \end{array}\right]+\left[\begin{array}{c} 0\\ b_{i} \end{array}\right]\tau_{i}$$

To understand the nonlinear embedding process, let us focus on the representation for Link 2. The COM simplified equation of movement for this link is given by

(27) $${\ddot{q}}_{2}=52.5\tau_{2}-52.5{\dot{q}}_{2}-49.3\cos q_{2}-5.28\sin q_{2}-0.016{\dot{q}}_{2}{\dot{q}}_{3}\sin2q_{3}$$

The extraction of the nonlinear embedded parameters then leads to:

(28) $$\theta_{2}\left(q\right)=\frac{-49.30495\cos q_{2}-5.27769\sin q_{2}}{q_{2}}$$

(29) $$\theta_{3}\left(q,\dot{q}\right)=-52.5-0.015876{\dot{q}}_{3}\sin\left(2q_{3}\right)$$

rendering the equation of movement as:

(30) $${\ddot{q}}_{2}=52.5\tau_{2}+\theta_{2}\left(q\right)q_{2}+\theta_{3}\left(q,\dot{q}\right)\dot{q}$$

Note than in order to perform the embedding for the $\theta_{2}$ parameter, a new division term has to be added. This is performed so that if (28) and (29) are substituted into (30), then we are back to the original nonlinear equation from (27). Thus, with (30), a pseudo-linear model is obtained that captures the full dynamics of the nonlinear models without losing information or generality as is the case when using linearization techniques.

For Link 1, the nonlinear embedding process leads to the following pseudo-linear representation:

(31) $$\left[\begin{array}{c} {\dot{q}}_{1}\\ {\ddot{q}}_{1} \end{array}\right]=\left[\begin{array}{cc} 0 & 1\\ 0 & \theta_{1}\left(q,\dot{q}\right) \end{array}\right]\left[\begin{array}{c} q_{1}\\ {\dot{q}}_{1} \end{array}\right]+\left[\begin{array}{c} 0\\ 10.75 \end{array}\right]\tau_{1}$$

where the nonlinear embedded parameters have the following complex and long expression given by:

(32) $$\begin{array}{c} \begin{array}{cc} \theta_{1}\left(q,\dot{q}\right)= & -10.75+0.081625{\dot{q}}_{2}\sin\left(2q_{2}\right)+0.003251{\dot{q}}_{2}\sin\left(2q_{2}\right)\cos{\left(q_{3}\right)}^{2}\\ \; & -0.0013887{\dot{q}}_{2}\cos{\left(q_{2}\right)}^{2}+0.00694{\dot{q}}_{2}\\ \; & +0.003251{\dot{q}}_{3}\sin\left(2q_{3}\right)\cos{\left(q_{2}\right)}^{2} \end{array} \end{array}$$

At this point, the second part of the LPV modelling comes into place, the polytopic representation of the system. The objective is to describe the new linear system on the varying parameters $\theta_{j}\left(q,\dot{q}\right)$ as a combination of linear systems, where each of these linear systems is the result of evaluating the new pseudo-linear systems (26) at the extreme values of the varying parameters $\theta_{j}\left(q,\dot{q}\right)=\left\{\underline{\theta_{j}},\bar{\theta_{j}}\right\}$. The combination of linear models, with $p=2^{n}$ vertexes, then forms the convex polytopic space description of the q-LPV (33) models for each *i* link of the gimbal.

(33) $$\left(\begin{array}{cc} A_{i}\left(\theta \right) & B_{i}\\ C_{i} & 0 \end{array}\right)=\sum_{k=1}^{p}h_{k}\left(\begin{array}{cc} A_{ik} & B_{i}\\ C_{i} & 0 \end{array}\right)$$

where

(34) $$\sum_{k=1}^{p}h_{k}=1$$

For the case of Link 1, whose LPV model is shown in (31), there is only one embedded parameter, so its polytopic space has two polytopic vertexes, shown in Table 1 and obtained by means of optimization using (32) as the objective function to be both minimized and maximized. By substituting those two values into the LPV representation, we obtain two state space linear systems that represent the polytopic vertexes of the qLPV model.

To obtain the polytopic model for Link 2, the analogue procedure is followed by finding the minimum and maximum values of each of the two embedded parameters. However, special care must be taken with the nonlinear embedded parameter $\theta_{2}\left(q\right)$, shown in (28), as it presents a discontinuity when the angular position becomes $q_{2}=0$ rad. To solve this problem, the solution is to divide the polytopic space for positive and negative values of the angular position. Thus, a hybrid controller will be required, which shows the versatility of the LPV approach. With this in mind, the extreme values of the embedded parameters which form the polytopic vertexes of the LPV model for Link 2 are shown in Table 2.

For Link 3, the LPV modelling is not required as after the COM simplifications its equations of movement are already linear.

(35) $$\left[\begin{array}{c} {\dot{q}}_{3}\\ {\ddot{q}}_{3} \end{array}\right]=\left[\begin{array}{cc} 0 & 1\\ 0 & -536.25 \end{array}\right]\left[\begin{array}{c} q_{3}\\ {\dot{q}}_{3} \end{array}\right]+\left[\begin{array}{c} 0\\ 536.25 \end{array}\right]\tau_{3}$$

## 4. Control Design Methodology

**Figure 7:**
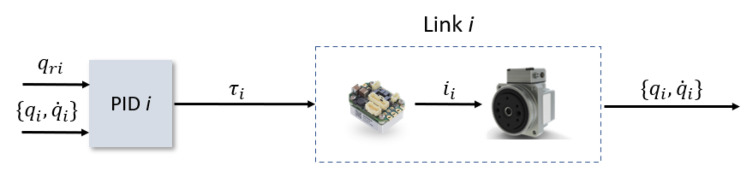
Control architecture for each Link *i*.

**Figure 8:**
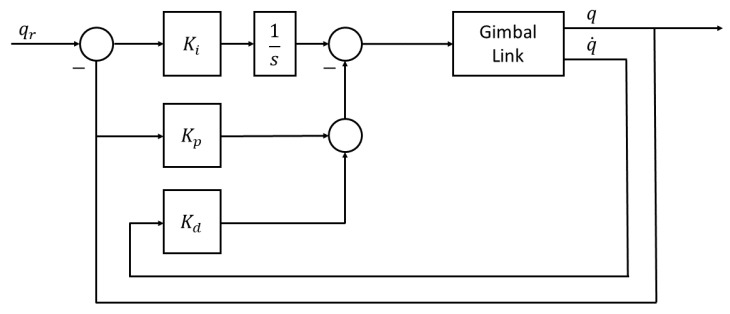
I-PD structure as implemented.

As previously mentioned, exploiting the low coupling interaction between the gimbal’s links, each link will have its own LPV model and as a result its own independent controller. In addition, since the objective of the three-axis gimbals is to fulfill a desired angular orientation, the reference variable for the control system is the angular position $q_{r}$ of each link. Meanwhile, the controlled variable will be the torque commands $\tau_{i}$ sent to each of the link’s servomotors. For the controller topology itself, given the system representation as seen in (26) and the reference variable, this problem presents a very good fit to the use of the PID LPV controllers [15]. The LPV bounding box method for LPV controller synthesis, based on assuring that all the vertexes of the qLPV polytope are considered during the synthesis step, will guarantee robustness against the nonlinearites of the system model, while the integrator action will guarantee the rejection of low frequencies disturbances and uncertainties that may affect the system. The PID architecture itself is an I-PD controller and takes advantages of the fact that all states are being measured with direct feedback from both the angular position and the angular velocity sensors, which is especially useful as it allows eliminating the use of a software derivative and with this, all the implementation issues that it has. The control architecture for each link can be seen in Figure 7 and the implemented structure of the PID in Figure 8.

The parameters of the PID will be computed using the polytopic gain-scheduling LPV approach. In order to achieve this, it is required to augment the second order system (26) that models each link by adding a new state $q_{e}$, which corresponds to the integral of the angular position error. The new augmented system can be seen in (36).  As can be seen in (37), the parameters of the PID controller are obtained from the coefficients of a state feedback controller, computed based on the new augmented state space representation of the system. However, using the LPV approach, we will not have a fixed PID controller but a family of controllers for each gimbal link

(36) $${\dot{x}}_{e}=\left[\begin{array}{ccc} 0 & 1 & 0\\ \theta_{j}\left(q,\dot{q}\right) & \theta_{j+1}\left(q,\dot{q}\right) & 0\\ 1 & 0 & 0 \end{array}\right]x_{e}+\left[\begin{array}{c} 0\\ 0\\ -1 \end{array}\right]q_{r}+\left[\begin{array}{c} 0\\ b_{i}\\ 0 \end{array}\right]\tau_{i}$$

(37) $$u=Kx_{e}=\left[-k_{p}-k_{d}-k_{i}\right]x_{e}$$

where

(38) $$x_{e}={\left[q\;\dot{q}\;\int \left(q-q_{r}\right)dt\right]}^{T}$$

Remember that the LPV polytopic representation allows modeling the system as a finite set of linear systems. By combining the polytopic representation with controller synthesis techniques based on Linear Matrix Inequalities (LMIs) [16], we can obtain a set of controllers, one for each vertex of our polytopic LPV model. This will ensure that the system will meet its design specifications, not only on the polytopic vertexes but within the whole polytopic space considered. This is what makes the LPV approach so powerful, as it allows for the use of well-known techniques from the linear control theory based on the Lyapunov theory of stability and LMIs optimization to be applied to nonlinear systems. This allows for a systematic approach for the design of nonlinear control systems that achieve tight design specifications, something which can only be achieved with other nonlinear control techniques after a long trial and error tuning process of the controller.

The LMIs are used as means to express the design objectives that we want our closed loop system to achieve. Examples of design objectives are the minimization of performance criteria as the $H_{2}$ and $H_{\infty }$ norms, and as in the case of this paper, it is also possible to impose pole placement as the goal for the control objective. What is powerful about this approach is the possibility to stack multiple LMIs to impose simultaneous performance objectives, or what is most interesting for the LPV approach, the possibility to include all of the polytopic vertexes. In this way, solving the LMI problem as a multi-objective optimization problem and including all the polytopic vertexes of the LPV model bounding box approach, the closed-loop system will achieve quadratic stability and the desired performance for the whole polytopic region [17] as long as a feasible solution $P=P^{T}$ to the following LMI problem exists:

(39) P>0

(40) $$A_{i}P+PA_{i}^{T}+BW_{i}+W_{i}^{T}B+2\alpha P<0$$

(41) $$M⊗\left(A_{i}P\right)+M^{T}⊗\left(PA_{i}^{T}\right)+M⊗\left(BW_{i}\right)+M^{T}⊗\left(W_{i}^{T}B^{T}\right)<0$$

Notice that ⊗ denotes the Kronecker product and

(42) $$M=\left[\begin{array}{cc} \sin\vartheta & \cos\vartheta \\ -\cos\vartheta & \sin\vartheta \end{array}\right]$$

**Figure 9:**
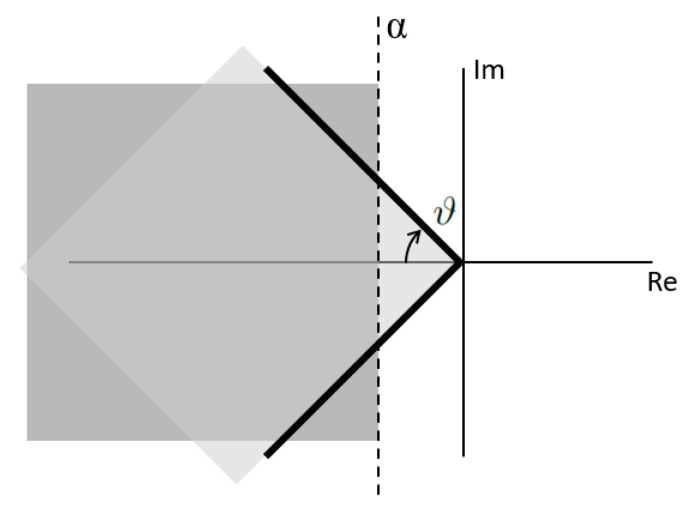
LMI regions in the pole space.

The LMIs used for the controller synthesis are collected in (39)–(41). The Lyapunov matrix *P* and the auxiliary control matrix $W_{i}$ serve as the decision variables. Equation (39) is used in order to guarantee stability according to the Lyapunov theory, which establishes that the Lyapunov matrix *P* must be positive defined. LMIs (40) and (41) are used as the design objectives to force the pole placement of the closed-loop system. Figure 9 shows how the parameters α and ϑ define the regions where the poles should be placed. With α, it is possible to control the rise time response and with ϑ the magnitude of the oscillations. The areas of intersection are where the poles will be placed if a solution to the LMI problem is feasible. In addition, notice that these equation present a sub-index *i*, meaning that for each one of the polytopic vertexes, a different pair of these two LMIs is required, thus covering the whole LPV polytopic space. The LMI problem can be formulated using the YALMIP [18] and solved by using the SeDuMi solver [19]. After a feasible solution is found, then the controller is computed as

(43) $$K_{i}=W_{i}P^{-1}.$$

Note here the use of the sub-index *i* means that for each polytopic vertex a different controller is computed. Then, in order to compute the controller to be implemented in real-time, the LPV gain-scheduling technique is used. The first step is to compute the scheduling function, which since for the three-axis gimbal case all of the polytopic regions have only two polytopic vertexes, it is quite straightforward and consists of a simple linear interpolation function

(44) $$h=\frac{\theta \left(q\right)-\underline{\theta }}{\bar{\theta }-\underline{\theta }}$$

Finally, the controller to be applied in real-time is computed according to

(45) $$K=\underline{K}\left(1-h\right)+\bar{K}h$$

Since the PID coefficients are extracted from the state feedback controller, it can be seen how the PID will adapt its coefficients with respect to the configuration of the gimbal link.

A second approach is to modify LMIs (40) and (41) so that instead of considering a unique auxiliary control matrix $W_{i}$ for each polytopic vertex we will have a global *W* matrix for all of the $A_{i}$ of the LPV model

(46) $$A_{i}P+PA_{i}^{T}+BW+W^{T}B+2\alpha P<0$$

(47) $$M⊗\left(A_{i}P\right)+M^{T}⊗\left(PA_{i}^{T}\right)+M⊗\left(BW\right)+M^{T}⊗\left(W^{T}B^{T}\right)<0$$

By doing this, we will be computing just one controller that must guarantee that all design objectives are fulfilled within the whole polytopic space. This approach is more conservative, but it is especially useful for Link 1, as can be seen from (32). Its embedded parameter has a long and complex expression. However, as can be seen from Table 1, the difference between the minimum and maximum value for its embedded parameter is small. This is a clear example for when it is a best decision to use a so-called polytopic LPV robust controller instead of the gain-scheduling LPV approach.

## 5. Virtual Sensor Fault Tolerant Control Methodology

In order to improve the robustness of the control system, the sensor redundancy present within each of the gimbal axes can be exploited. Remember that the reference gimbal has a dedicated sensor for measuring the angular position and a second one for measuring the angular velocity although both sensors could be used for measuring both variables. However, with a configuration in which each sensor works independently, a failure of one of them will cause the failure of the whole control system. In order to prevent the loss of control performance in the case of a sensor fault and thanks to the given sensor redundancy, a good fault tolerant control (FTC) strategy is to use a virtual sensor [20].

**Figure 10:**
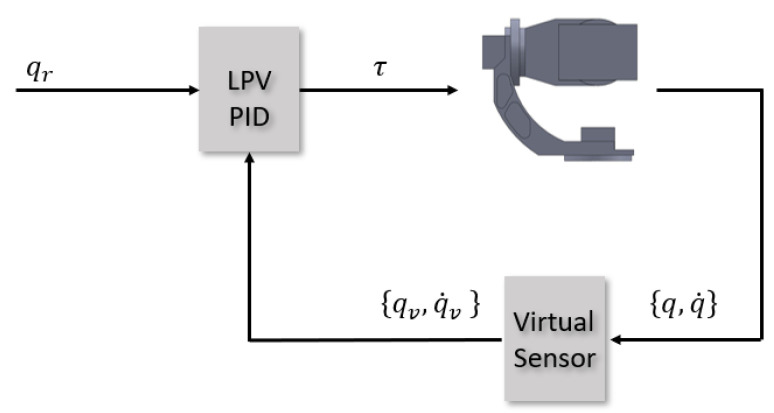
Closed-loop control system for the three-axis gimbal with virtual sensor incorporated.

Figure 10 shows how the closed-loop control system looks when the virtual sensor strategy is incorporated. As can be seen, the inputs to the virtual sensor are the sensor readings, which may be faulty, and the outputs are the corrected sensors readings that are used as the feedback signal to the controller. When compared with active fault tolerant control, the benefit of the virtual sensor strategy resides in the fact that the sensor fault tolerance is achieved without requiring modification to the nominal controller itself. Hence, the virtual sensor guarantees that the original controller will continue to perform normally in the sensor fault scenario.

When considering sensor faults for LPV systems [21], the model can be expressed in the following way:

(48) $$\begin{array}{c} \begin{array}{cc} \; & \dot{x}=A\left(\theta \right)x+B\left(\theta \right)u\\ \; & y_{f}=C\left(\theta ,\gamma \right)x+f_{y} \end{array} \end{array}$$

where the *C* matrix may be a function of the embedded varying parameters θ and the multiplicative faults γ

(49) $$C\left(\theta ,\gamma \right)=diag\left(\gamma_{1},\gamma_{2},\cdots ,\gamma_{n}\right)C\left(\theta \right)$$

Additionally, the sensor output equation may be affected by a vector of additive reading faults $f_{y}$. For the case of the reference three-axis gimbal model, both *B* and *C* matrices are parameter independent. Furthermore, since both model states can be measured with dedicated sensors, the *C* matrix is 2×2 identity matrix. Its output sensor equation when considering sensors faults is:

(50) $$\left[\begin{array}{c} q_{yf}\left(k\right)\\ {\dot{q}}_{yf}\left(k\right) \end{array}\right]=\left[\begin{array}{cc} \gamma_{1}\left(k\right) & 0\\ 0 & \gamma_{2}\left(k\right) \end{array}\right]\left[\begin{array}{cc} 1 & 0\\ 0 & 1 \end{array}\right]\left[\begin{array}{c} \hat{q}\left(k\right)\\ \hat{\dot{q}}\left(k\right) \end{array}\right]+\left[\begin{array}{c} f_{y1}\left(k\right)\\ f_{y2}\left(k\right) \end{array}\right]$$

From the modified sensor output equation to the case of sensor faults (50), it can be seen that in the nominal situation the multiplicative fault parameter should have a value γ(k)=1, while the additive fault parameter should have a value $f_{y}\left(k\right)=0$. Notice also that both states from the state vector present a hat symbol, representing that the real fault-free states are assumed to be known.

To build the virtual sensor for LPV systems, its structure will depend on the following condition

(51) rank(C(θ,γ))=rank(C(θ))≠0

which for the gimbal system will depend only on the value of the additive fault γ. So, for the cases where only partial sensor faults are occurring γ≠0, no total signal loss from the sensor condition (51) will be satisfied. In this case, the structure of the virtual sensor for LPV systems is given by the following static gain corresponding to the model matching reconfiguration

(52) $$y_{v}=C\left(\theta \right)C{\left(\theta ,\gamma \right)}^{-1}\left(y_{f}-f_{y}\right)$$

For the gimbal system, and focusing only on the sensor reading for the angular position for the sake of simplicity, the static gain that defines the virtual sensor equation according to (52) is defined by

(53) $$q_{v}\left(k\right)=\frac{q_{yf}\left(k\right)-f_{y1}\left(k\right)}{\gamma_{1}\left(k\right)}$$

In the case where condition (51) is not satisfied, total loss of a sensor signal or more, then the virtual sensor would consist of a state observer of the system using the remaining healthy sensors. However, only partial sensor faults are considered in this paper.

The virtual sensor (53) is just a static gain. However, it assumes the online knowledge of the multiplicative and additive fault coefficients. Then, these values should be obtained by means of a real-time estimation for both values γ(k) and $f_{y}\left(k\right)$. In order to solve the estimation problem in an efficient form for real-time implementation of the virtual sensor, the Recursive Least Square (RLS) estimation method can be used. To use the RLS algorithm, the estimation variables relation should be formulated in the parameter identification regressor form

(54) $$y\left(k\right)=\varphi^{T}\left(k\right)\Theta \left(k\right)+e\left(k\right)$$

Then, according to the regressor Equation (54), the output sensor Equation (50) for the individual angular position sensor can be rearranged as:

(55) $$q_{yf}\left(k\right)=\left[\begin{array}{cc} \hat{q}\left(k\right) & 1 \end{array}\right]\left[\begin{array}{c} \gamma (k)\\ f_{y}\left(k\right) \end{array}\right]$$

where the output of the regressor is the faulty measurement from the sensor $y\left(k\right)=q_{yf}\left(k\right)$, the regressor vector consisting of known values is $\varphi^{T}\left(k\right)=\left[\hat{q}\left(k\right)\;1\right]$, and the vector of estimated variables is $\Theta \left(k\right)={\left[\gamma \left(k\right)\;f_{y}\left(k\right)\right]}^{T}$. The term, e(k) is the identification error and is expected to be zero. The identification error can be computed based on the parameter estimation from the previous sample:

(56) $$e\left(k\right)=y\left(k\right)=\varphi^{T}\left(k\right)\Theta \left(k-1\right)$$

Then, in order to update the parameters estimation, the RLS gain vector should be updated according to:

(57) $$K\left(k\right)=\frac{P(k-1)\varphi (k)}{\lambda +\varphi^{T}\left(k\right)P\left(k\right)\varphi \left(k\right)}$$

and the covariance matrix according to:

(58) $$P\left(k\right)=\frac{1}{\lambda }\left[P\left(k-1\right)-\frac{P\left(k-1\right)\varphi \left(k\right)\varphi^{T}\left(k\right)P\left(k-1\right)}{\lambda +\varphi^{T}\left(k\right)P\left(k\right)\varphi \left(k\right)}\right]$$

Then, the parameter estimation should be updated as

(59) Θ(k)=Θ(k−1)+K(k)e(k)

The parameter λ is known as the forgetting factor and is a design parameter used to allow the parameter estimation to track changes in the parameter values by controlling the effect that old data has on the estimation that may not be relevant anymore. Usual values for λ are in the range [0.9,1], and its choices affect the batch of $N=\frac{2}{1-\lambda }$ data used in the estimation.

Algorithm 1 summarises the online fault estimation using the RLS method.
**Algorithm 1:** Recursive Least Square Algorithm.**Input**: q(k),$\hat{q}\left(k\right)$**Output**: λ(k),$f_{y}\left(k\right)$Compute the identification error by using (56).Update the gain vector with (57) and the covariance matrix with (58).Update the estimated parameters by applying (59).

It should be noted that the results shown in the following section were obtained using the RLS block from the Simulink library with a forgetting factor of λ=0.9. The use of this block has the advantage that it supports automatic code generation in C/C++ for practical implementations of the virtual sensor FTC algorithm.

From the description on (55), note that the fault-free state is assumed to be a known variable. In order to obtain its value, an LPV observer is used; hence the use of the hat symbol. It is well-known that in a state–feedback design a controller–observer duality exists, in which applying the following transformations during the controller gain *K* synthesis

(60) $$A⟹A^{T}=\tilde{A}$$

(61) $$B⟹C^{T}=\tilde{B}$$

leads to obtaining an observer gain *L* by applying the final transformation

(62) $$L=K^{T}$$

Exploiting this duality, LPV observers can be designed by substituting the transformations (60) and (61) in the set of design LMIs (39)–(41) as the new *A* and *B* variables. So, by solving the new convex LMIs optimization problem with $\tilde{A}$ and $\tilde{B}$, a set of observers gains *L* can be computed for each of the gimbal links. Exactly as in the case of the controller design, the observer gain reconstruction is performed via linear interpolation using (44) and (45). Finally, the LPV observer is implemented as a classic Luenberger observer

(63) $$\dot{\hat{x}}=A\left(\theta \right)\hat{x}+B\tau -L\left(\theta \right)\left(y-C\hat{x}\right)$$

In the observer Equation (63), A(θ) could be computed either as a linear interpolation by using the same procedure as (44) and (45) or by direct substitution of the varying parameters θ in the LPV model (26).

An important remark in the design of the LPV observer for this particular virtual sensor application is that in order for the FTC block to have the expected performance, it is required to decouple the state observer dynamics from the fault parameter estimation.

**Figure 11:**
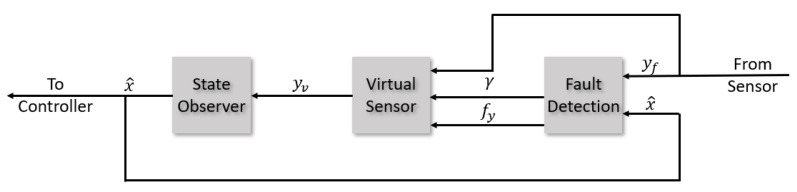
Virtual sensor fault tolerant block scheme.

This remark is evident by inspecting the whole FTC block, Figure 11, now that all the elements have been presented. It can be seen that there is an important coupling between the elements that forms the virtual sensor. The inputs for the LPV state observer comes from the virtual sensor Equation (53) that depends on the additive γ and multiplicative $f_{y}$ fault parameters detected by the recursive least square online estimation, which in turn uses the state prediction $\hat{x}$ from the LPV observer as the regressor input. The virtual sensor equation is a static expression. As a result, the coupling in the virtual sensor block comes from the dynamics of the LPV observer and the RLS fault estimator. To achieve the decoupling, we force one of them to have its dynamics be many orders of magnitude faster than the other. As the focus of the virtual sensor is on fault tolerance and fault detection, the element with the fastest dynamics corresponds with the RLS fault estimator. This is achieved by selecting an aggressive forgetting factor of 0.9, as previously introduced, and by simultaneously forcing the LPV observer to have slow convergence dynamics, which can be accomplished by setting the decay rate α at the design LMI (40) to a low value.

Forcing the observer dynamics to be slow can also have an intuitive interpretation as if from the control designer point of view we want our observer to have a high level of “inertia” so that in the presence of a sensor fault it will not converge immediately to the new faulty sensor signal and instead remain in the “nominal state path”. This allows the RLS estimator to be much faster to detect the fault parameters, and then the virtual sensor equation can reconstruct the non-faulty sensor signal.

## 6. Results

### 6.1. Simulation Scenarios

The resulting control system is tested for a given trajectory in a series of different scenarios: first, in the nominal situation and then twice in a scenario where there are sensor faults occurring in the angular position sensor. In the case where the angular position sensor is faulty, the effects of the sensor fault in a control system without any FTC strategy are first shown, and then a second simulation highlights the benefits of the virtual-sensor-based fault tolerant strategy presented in this work. Note that for the sake of simplicity and clarity, results from faults on the angular speed sensor and plots for Link 3 have been avoided as they will not add any new insight into the results. For the case of Link 3, it was also seen that its COM is a simple linear plant, so its control is not a great challenge anyway. However, this could also be interpreted as if $q_{3ref}=0$, which is equivalent to saying that we want the mounted camera to be parallel to the horizontal plane at all time, which is a reasonable assumption.

In addition in order to make the simulations as realistic as possible, the tested SOM is from (20) which introduces the effect of the Coulomb frictions neglected during the controller synthesis. Additionally, the parameters for Link 3 have been modified in the SOM to assume that there is a camera mounted as an end-effector of the three-axis Gimbal. These modifications are covered in the Appendix A.

**Figure 12:**
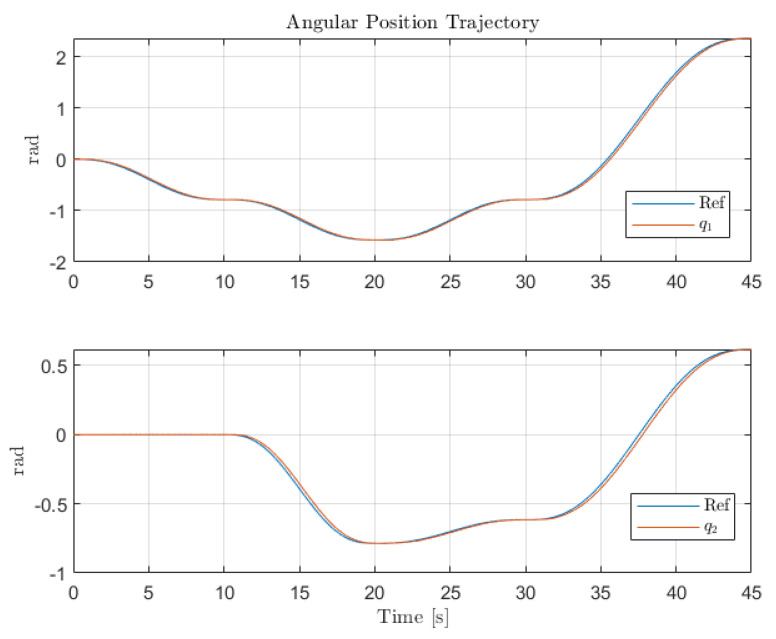
Individual trajectories for $q_{i}$ of Links 1 and 2.

**Figure 13:**
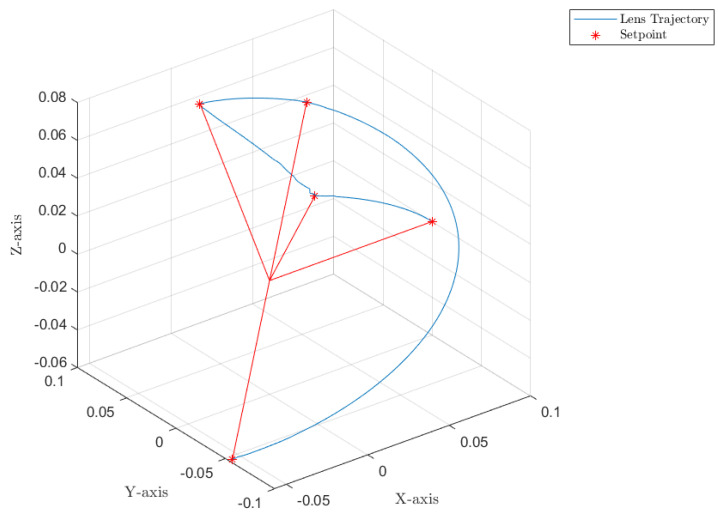
Combined trajectories of Links 1 and 2 to form the camera path in $R^{3}$ Cartesian coordinates.

**Figure 14:**
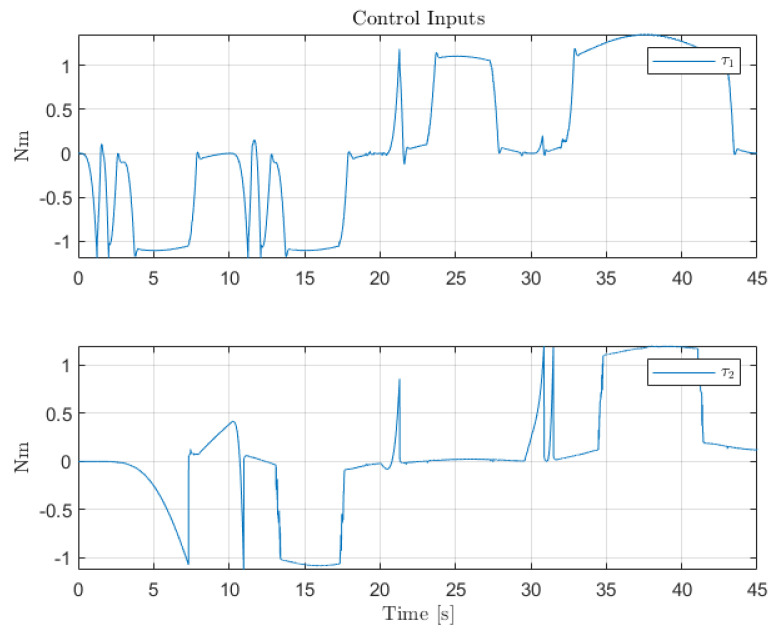
Control inputs for the servo-actuators of Links 1 and 2.

**Figure 15:**
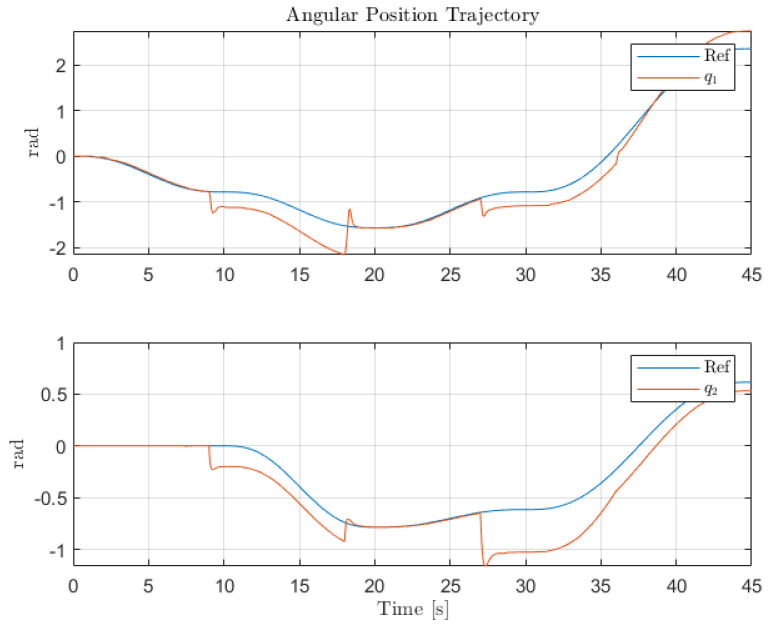
Individual trajectories for $q_{i}$ of Links 1 and 2.

**Figure 16:**
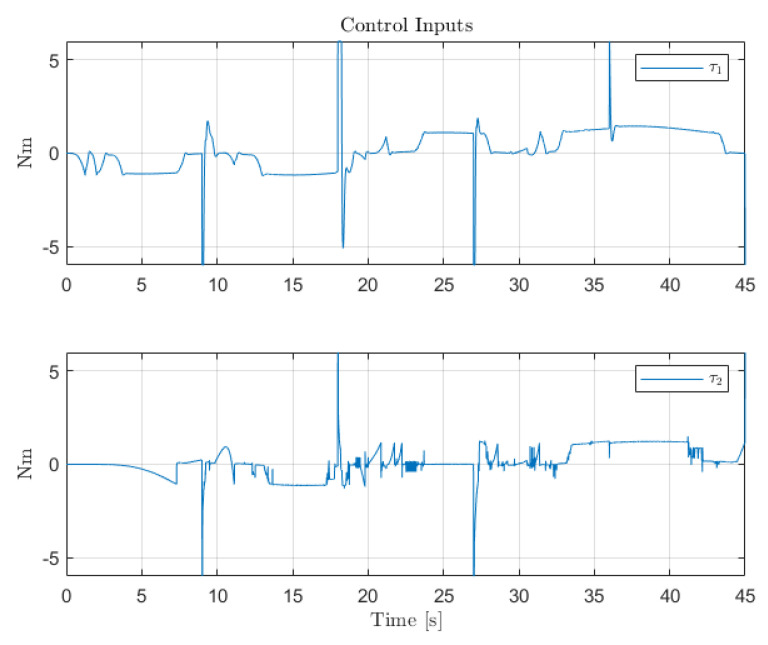
Control inputs for the servo-actuators of Links 1 and 2.

**Figure 17:**
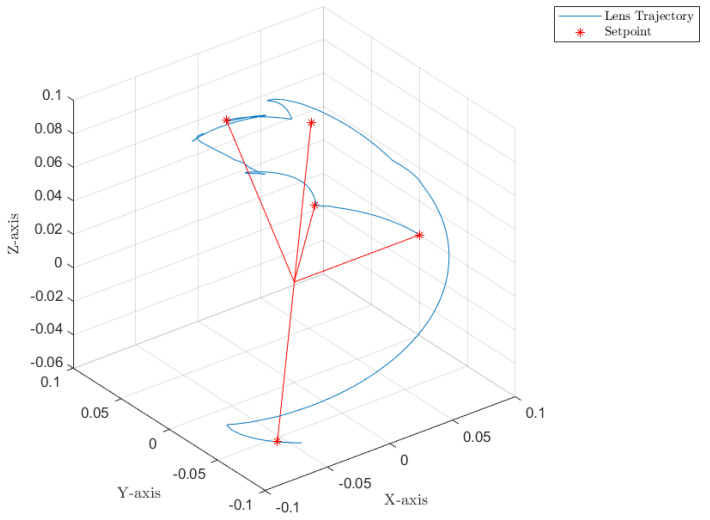
Combined trajectories of Links 1 and 2 to form the camera path in $R^{3}$ Cartesian coordinates.

**Figure 18:**
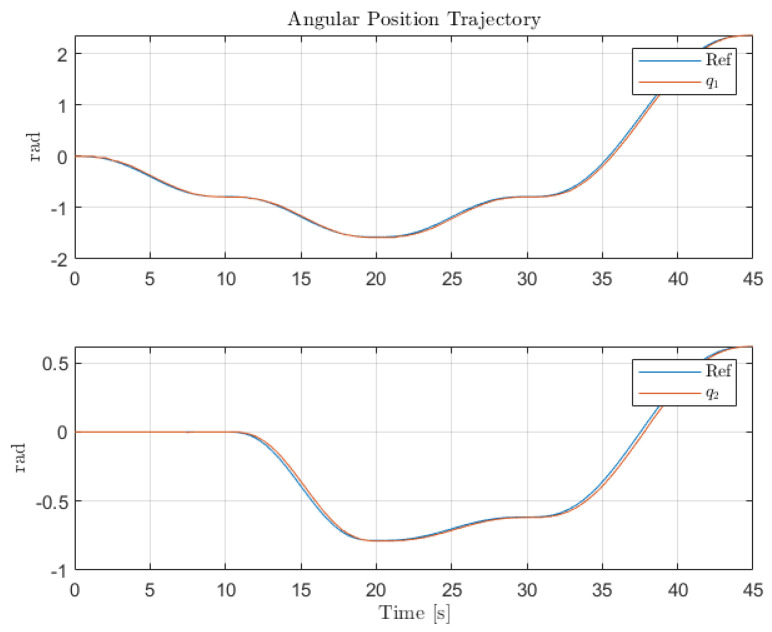
Individual trajectories for $q_{i}$ of Links 1 and 2.

**Figure 19:**
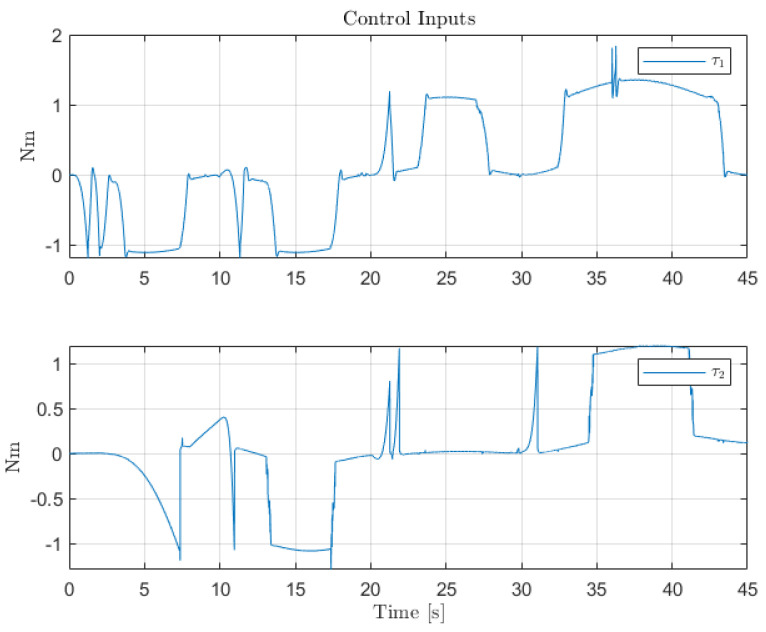
Control inputs for the servo-actuators of Links 1 and 2.

**Figure 20:**
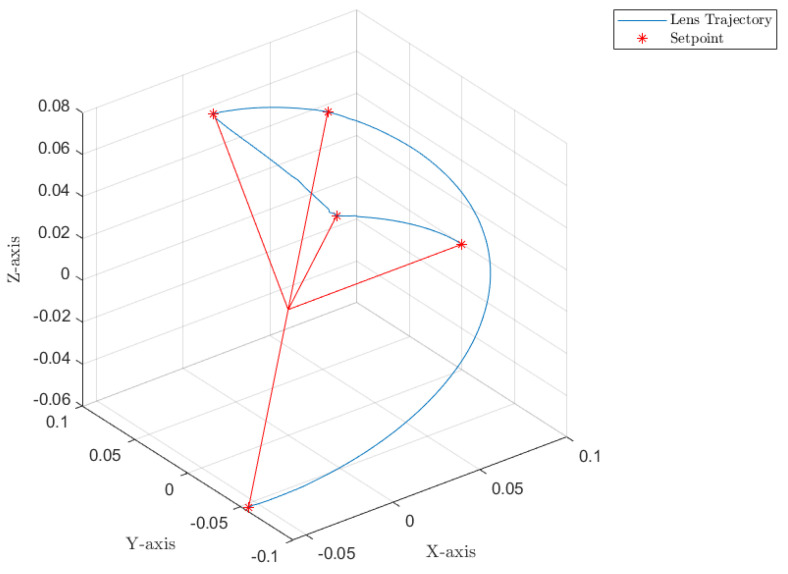
Combined trajectories of Links 1 and 2 to form the camera path in $R^{3}$ Cartesian coordinates.

**Figure 21:**
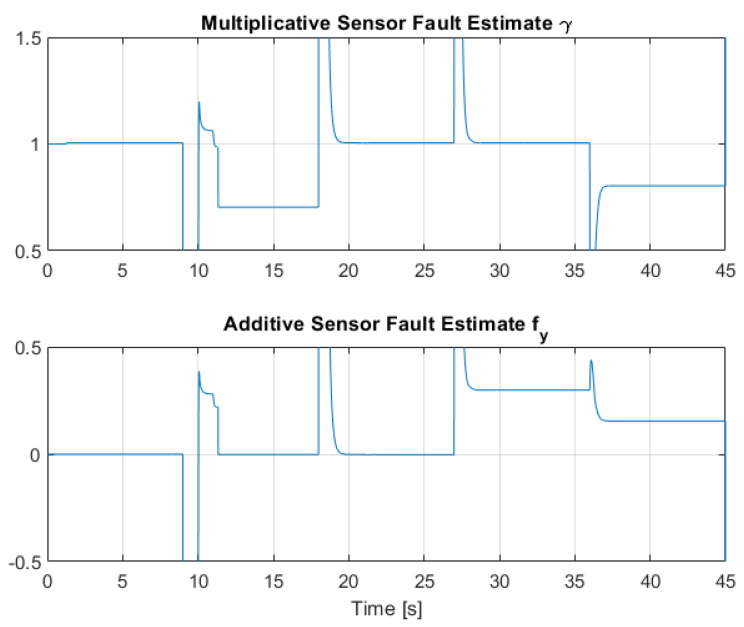
Multiplicative and additive sensor fault estimation by means of RLS for Link 1.

**Figure 22:**
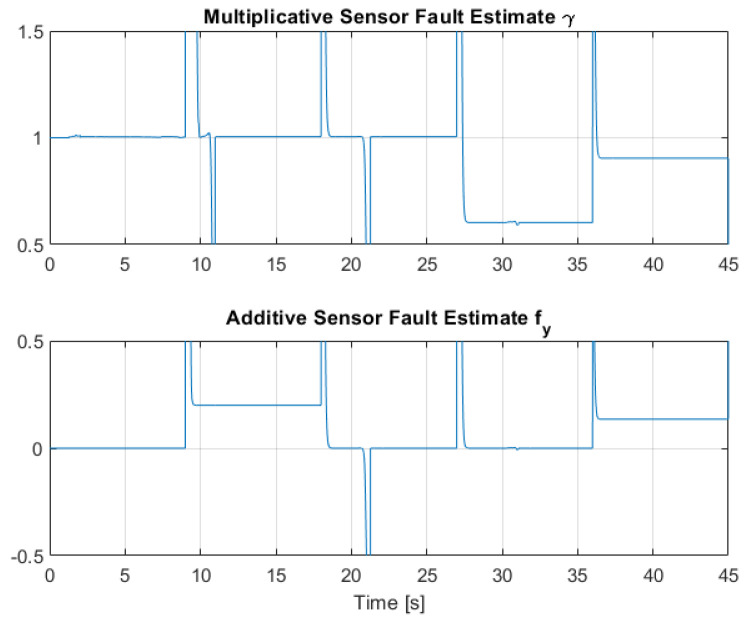
Multiplicative and additive sensor fault estimation by means of RLS for Link 2.

Figure 12, Figure 13 and Figure 14 collect the results from testing the designed control system against the SOM in the nominal situation, meaning no sensor fault but with the modified SOM including the mounted camera. In Figure 15, Figure 16 and Figure 17, the simulation results in the presence of faulty sensors are collected when the original control scheme is considered without virtual sensors. Finally, Figure 18, Figure 19, Figure 20, Figure 21 and Figure 22 collects the results when the PID LPV controller plus the virtual sensor fault tolerant strategy are used in conjunction.

In the faulty scenarios, the applied faults for Links 2 and 3 are given in the following Table 3 and Table 4:

### 6.2. Discussion on Results

The first scenario represents the nominal control of the three-axis gimbal according to the control methodology presented in Section 4 in the absence of sensor faults. Figure 12 shows the reference trajectory decomposed in its $q_{ir}$ elements and the tracking performance of each individual link as well. It can be clearly seen that the designed LPV-PID controllers are more than able to cope with the nonlinearities present in the three-axis gimbal system and are also robust against low frequency uncertainties and disturbances, e.g., the mass change of Link 3 of the SOM in comparison with the COM or the effects of the Coulomb frictions. It can also be seen in Figure 14 that the control system is able to fulfill a pretty good tracking performance while having a controller signal totally within the nominal parameters of the servo actuators used in the reference gimbal. Finally, Figure 13 shows the combined trajectories of Links 1 and 2 in world space coordinates in the gimbal base frame, simulating the path of a mounted camera and with the path normalized to a sphere of radius 0.1 meters. The translation from link joint frame $q_{i}$ to world space coordinates is performed using the IK algorithm introduced earlier. In this figure, the set-points from the given world space Cartesian trajectory can also be seen, and it shows that the gimbal path fulfills this requirement quite well.

The second scenario shows the performance of the original control structure in the presence of sensor faults. As expected, without the presence of any FTC component, the closed-loop performance is greatly affected by the presence of the faults in the angular position sensors for Links 1 and 2. It should be noted that since only partial sensor faults are considered, the system does not lose stability. However, the sudden changes of sensors reading the LPV-PID experience produce controller signals that cause the saturation of the actuators. Thus, this shows that sensor faults not only hinder the closed-loop performance but also the integrity and the lifespan of the system.

The third scenario deals with the case where the presence of sensor faults is greatly mitigated by the presence of the Virtual Sensor methodology introduced in Section 5. The results show that the virtual sensor block performance is very good, as this combined sensor fault case is the same as the nominal case regarding the closed-loop response and tracking performance. Thus, the virtual sensor is fully capable of masking the sensor faults to the nominal PID LPV controller, and the system is capable of robust performance against partial sensor malfunctions. Figure 21 and Figure 22 show the multiplicative γ and additive $f_{y}$ fault parameter estimation by the RLS regression estimator. It can be observed that the estimation effectively converges to the applied faults shown in Table 3 and Table 4.

## 7. Conclusions

In this paper, it was shown how to obtain accurate analytical models for three-axis gimbal systems by using the Serial-Link theory from the robotics field. In addition, a series of simplifications that can be applied to the complex analytical model by taking advantage of the unique mechanical design on the construction of gimbal systems was demonstrated via open-loop simulation. These simplifications allow us to obtain a new model description which is suitable for translating the nonlinear equation of movement of the gimbal into three decoupled polytopic qLPV models. The modelling procedure was shown for a reference gimbal as an example. However, the modelling steps could be easily extended to obtain LPV models for all kind of three-axis gimbal systems. Moreover, the analytical model based on Lagrangian methods is very generic and can be applied with little modification to other gimbal systems.

In addition, to provide a systematic approach for obtaining qLPV models for three-axis gimbal systems, it was shown how a polytopic LPV model can be used to synthesize feedback controllers was shown. Using well-established LMI methods based on Lyapunov theory, pole placement specifications for a nonlinear system extending classic linear control approaches were acquired. Given the links model structure and having angular position tracking as the control objective, the LPV models were augmented to obtain PID LPV controllers. The controllers proved to be adaptive to the nonlinearites in the whole configuration range of the gimbal while giving robustness against low-frequency disturbances and uncertainties that may be present in the system. Concretely, it was shown via simulation that despite modifying the SOM to simulate a mounted camera, the control system was capable of good tracking performance.

Finally, it was shown that in systems with sensor redundancy, as in the reference three-axis gimbal, this sensor redundancy can be exploited by means of virtual sensors to obtain robustness against partial sensor faults. The implemented fault tolerant strategy works by decomposing the output sensor equation in such a way that multiplicative and additive sensor faults are taken into consideration. As a result, these parameters can be estimated by using online recursive regression. For the completion of the virtual sensor algorithm, an LPV observer was synthesized by simply exploiting the well known controller–observer duality for state-feedback control design. Given that the virtual sensor algorithm relies on RLS and a LPV Luenberger observer, its practical implementation does not pose a challenge, which is quite remarkable given the demonstrated performance.

In future works, we expect to implement the LPV control solution and the virtual sensor strategy presented here in a real three-axis gimbal system. Additionally, exploiting the sensor redundancy information presented in the reference gimbal system, an interesting research problem is to extend the fault tolerant capabilities presented here by also considering the possibility of incipient hardware faults [22] in the electric motors that operate each of the gimbal axes. In particular, in addition to the position and speed sensors considered in this work, available sensor information from the servomotors for voltage and current measurement can be used for the detection of motor faults. 

## Figures and Tables

**Table 1:** Link embedded parameter max. and min. value.

Parameter	Value
$\underline{\theta_{1}}$	−11.2847
$\bar{\theta_{1}}$	−10.2153

**Table 2:** Link 2 embedded parameters max. and min. value.

Parameter	Value
$\underline{\theta_{2}}$ ($q_{2}$ negative)	−17.3492
$\bar{\theta_{2}}$ ($q_{2}$ negative)	485.3176
$\underline{\theta_{2}}$ ($q_{2}$ positive)	−495.8560
$\bar{\theta_{2}}$ ($q_{2}$ positive)	16.0752
$\theta_{3}$	≈−52.5

**Table 3:** Sensor faults applied in $q_{1}$ for the faulty scenarios.

	0–9	9–18	18–27	27–36	36–45
** γ **	1	0.7	1	1	0.8
** $f_{y}$ **	0	0	0	0.3	0.2

**Table 4:** Sensor faults applied in $q_{2}$ for the faulty scenarios.

	0–9	9–18	18–27	27–36	36–45
** γ **	1	1	1	0.6	0.9
** $f_{y}$ **	0	0.2	0	0	0.15

## Data Availability

All the required data is included in the paper.

## Appendix A. Three-Axis Gimbal Parameters

### Appendix A.1. Motor Parameters

Maximum torque 8.3 Nm, Allowable continuous current 3.5 A, Conversion torque constant 1.3 Nm/A.

### Appendix A.2. Direct Kinematics

**Table A1:** DH Parameters for the Three-Axis Gimbal.

Link	$\theta_{i}$	$a_{i}$	$d_{i}$	$\alpha_{i}$
1	$q_{1}^{*}+\pi /2$	0	0	π/2
2	$q_{2}^{*}-\pi /2$	0	0	−π/2
3	$q_{3}^{*}$	0	0	0

* variable.

**Table A2:** DH Parameters relating the base frame to reference frame of Link 1.

Link	$\theta_{i}$	$a_{i}$	$d_{i}$	$\alpha_{i}$
Base	π/2	0	0	π

### Appendix A.3. Mechanical Properties of the Links

   Link 1:

$m_{1}$ = 2.492 kg, Center of Mass (CoM) = [−0.0906, 0, 0.1147] m, Inertia tensor elements
$I_{xx}=0.0144$ Kg·m${}^{2}$$I_{yy}=0.0294$ Kg·m${}^{2}$$I_{zz}=0.0175$ Kg·m${}^{2}$ $I_{xz}=0.0175$ Kg·m${}^{2}$

   Link 2:

$m_{2}$ = 1.624 kg, CoM = [−0.0063, 0, −0.0734] m, Inertia tensor elements $I_{xx}=0.0090$ Kg·m
${}^{2}$$I_{yy}=0.0085$ Kg·m${}^{2}$$I_{zz}=0.0020$ Kg·m${}^{2}$$I_{xz}=-0.0007$ Kg·m${}^{2}$

   Link 3:

$m_{3}$ = 0.756 kg, CoM = [0, 0, −0.0310] m, Inertia tensor elements $I_{xx}=0.0013$ Kg·m
${}^{2}$$I_{yy}=0.0006$ Kg·m${}^{2}$$I_{zz}=0.0019$ Kg·m${}^{2}$

   Modified Link 3 for the SOM:

$m_{3}$ = 2.106 kg, CoM = [0, 0, −0.0530] m, Inertia tensor elements $I_{xx}=0.0033$ Kg·m ${}^{2}$
$I_{yy}=0.0027$ Kg·m${}^{2}$$I_{zz}=0.0041$ Kg·m${}^{2}$

   Viscous Friction:

$F_{v}=1$

   Coulomb Friction:

$\mu_{k}=0.8$, $F_{s}=1.2$ Nm
